# Comparison of Methods for Isolation of Bacterial and Fungal DNA from Human Blood

**DOI:** 10.1007/s00284-013-0451-1

**Published:** 2013-09-12

**Authors:** Tomasz Gosiewski, Leszek Szała, Agata Pietrzyk, Monika Brzychczy-Włoch, Piotr B. Heczko, Małgorzata Bulanda

**Affiliations:** 1 Chair of Microbiology, Jagiellonian University Medical College, 18 Czysta Str., 31-121 Kraków, Poland; 2 Institute of Mathematics, Silesian University, 746 01 Opava, Czech Republic

## Abstract

The study aimed at optimization of DNA isolation from blood of representatives of four microbial groups causing sepsis, i.e., Gram negative: *Escherichia coli*, Gram positive: *Staphylococcus aureus,* yeast: *Candida albicans*, and filamentous fungus: *Aspergillus fumigatus*. Additionally, the five commercial kits for microbial DNA isolation from the blood were tested. The developed procedure of DNA isolation consisted of three consecutive steps, i.e., mechanical disruption, chemical lysis, and thermal lysis. Afterward, DNA was isolated from the previously prepared samples (erythrocyte lysis) with the use of five commercial kits for DNA isolation. They were compared paying heed to detection limit, concentration, DNA purity, and heme concentration in samples. The isolation of DNA without preliminary erythrocyte lysis resulted in far higher heme concentration than when lysis was applied. In the variant with erythrocyte lysis, two of the commercial kits were most effective in purifying the DNA extract from heme. Designed procedure allowed obtaining microbial DNA from all four groups of pathogens under study in the amount sufficient to conduct the rtPCR reaction, which aimed at detecting them in the blood.

## Introduction

Effective diagnostic of factors causing the infection is the most important, and most difficult, problem in the treatment of blood infections. It is decisive as regards the effectiveness of therapy and, consequently, the costs and the duration of hospitalization. The identification of etiological factors allows to employ efficient targeted antibiotherapy. The material submitted for the study is blood taken from patients manifesting clinical symptoms of sepsis. Until now, the so-called diagnostic “gold standard” has been constituted by blood cultures carried out on special growth media, preferably in automated cell culture systems. The advantages of such methods are their simplicity and relatively low costs of testing. Their weakness is that they are time-consuming, taking up to 5 days (until the test results are issued), and have low detection limit, which causes only 15–20 % of the culture to obtain microbial growth [3].

In order to enhance the chances of identifying microbes in blood, attempts are undertaken to identify them with the help of nucleic acid detection. The difficulty lies in isolating a proper quality of DNA matrix. It is necessary for it to be of the highest possible concentration. Fungi and different species of bacteria, which may be factors inducing sepsis (sometimes polietiological), are characterized by varied susceptibility to cell lysis and, consequently, the possibility of obtaining DNA from them may also vary.

In scientific literature, there is a lack of a description of a method for isolating DNA from blood which would be effective in the case of both bacteria and fungi. Many authors present methods which boil down to either isolating eukaryotic DNA from leukocytes or separately—from bacteria or from fungi [1, 2, 8]. There have been ready-to-use kits available on the market for several years now, e.g., Septifast (Roche) or Septitest (Molzym), serving the purpose of detecting microbes in blood; however, the need for working on new solutions is still in order [5, 10].

The objective of the study was to develop a comprehensive method for microbial (bacterial and fungal) DNA isolation from blood to detect these pathogens and to evaluate the usefulness of commercial kits for DNA isolation.

## Materials and Methods

### Microbial Strains

Four strains were used in the research, these were representing groups diversified as regards the cell wall structure: Gram-negative *Escherichia coli* ATCC 25922 (American Type Culture Collection), Gram-positive *Staphylococcus aureus* ATCC 33497, *Candida albicans* ATCC 10231 yeast, and *Aspergillus fumigatus* ATCC 14110 filamentous fungus.

### Blood Samples

Blood was collected from volunteers, who had no clinical symptoms of sepsis and no inflammatory markers (CRP and OB). Blood samples were drawn into 2-mL Vacutainer K_3_E (Becton–Dickinson) test tubes. The research was granted approval by the local Bioethics Committee of the Jagiellonian University (KBET/94/B/2009).

### The Method for Microbial DNA Isolation from Blood

DNA was isolated from 1.5-mL blood samples, which were simultaneously inoculated with four model microbial strains (*E. coli*, *S. aureus*, *C. albicans*, and *A. fumigatus*), in order to obtain the cell number of 10^6^ CFU/mL for each of them. The number of the *A. fumigatus* conidia was determined by counting in a Bürker chamber.

The standardization of the method consisted in testing different variants of preliminary blood samples processing: (a) the samples were subjected to erythrocyte lysis in 0.17 M ammonium chloride solution (Sigma) (at the same time, DNA was isolated to the exclusion of this stage, in order to compare results); (b) mechanical disruption was employed in the presence of glass beads ϕ 710–1,180 μm (Sigma) in a FastPrep machine (MP Biomedicals); (c) enzymatic lysis of bacteria was conducted in the presence of the following enzymes: lysozyme (2 mg/mL), lysostaphin (0.2 mg/mL), and lyticase 40 U (Sigma) at the temperature of 37 °C; and (d) the samples were subjected to 50 mM NaOH (POCh) at 95 °C.

In the next stage, DNA was isolated from the samples using commercial kits for DNA isolation (following the manufacturers’ protocols which included lysis of leukocytes and proteins in the samples), in order to select the optimal one: GeneMATRIX Quick Blood DNA Purification Kit (EURx); GeneJET (Fermentas); QIAamp DNA Blood (QIAGEN); Blood Mini (A&A Biotechnology); and Genomic Mini (A&A Biotechnology). DNA was eluted from the columns with the use of 100 μL Tris buffer. In the case of each set, DNA was isolated from 16 samples.

### DNA Amplification

All the processes of DNA amplification were performed with the use of the real-time PCR method (rtPCR) in a thermocycler CFX96 (BioRad) by employing species-specific primers and TaqMan probes. The sequences of oligonucleotides utilized in the research and amplification procedures are presented in Table 1.

**Table 1:** Oligonucleotide sequences employed in DNA amplification reactions of the four microbes

Microbe species	5′–3′	Amplification procedure
Gram (−) *E. coli*	(F) GGGAGTAAAGTTAATACCTTTGC (R) CTCAAGCTTGCCAGTATCAG FAM-CGCGATCACTCCGTGCCAGCAGCCGCGGATCGCG-BHQ1 [2]	95 °C for 2 min (95 °C for 15 s, 55 °C for 30 s, 72 °C for 30 s) 50 cycles
Gram (+) *S. aureus*	(F) TACATGTCGTTAAACCTGGTG (R) TACAGTTGTACCGATGAATGG (P) FAM-CGCGATCCAAGAACTTGTTGTTGATAAGAAGCAACCGATCGCG-BHQ1 [2]	95 °C for 2 min (95 °C for 15 s, 61 °C for 30 s, 72 °C for 30 s) 50 cycles
*C. albicans* yeast	(F) TTGGTGGAGTGATTTGTCTGCT (R) TCTAAGGGCATCACAGACCTG (P) FAM-TTAACCTACTAAATAGTGCTGCTAGC-BHQ1 [8]	95 °C for 2 min (95 °C for 15 s, 55 °C for 30 s, 72 °C for 30 s) 50 cycles
*A. fumigatus* filamentous fungus	(F) TTGGTGGAGTGATTTGTCTGCT (R) TCTAAGGGCATCACAGACCTG (P) FAM-TCGGCCCTTAAATAGCCCGGTCCGC-BHQ1 [8]	95 °C for 2 min (95 °C for 15 s, 61 °C for 30 s, 72 °C for 30 s) 50 cycles
β-actin gene	(F) GCCAGTGCCAGAAGAGCCAA (R) TTAGGGTTGCCCATAACAGC [9]	95 °C for 5 min (95 °C for 30 s, 55 °C for 30 s, 72 °C for 1 min) 30 cycles and final extension at 72 °C for 5 min

*F* forward primer, *R* reverse primer, *P* probe

Additionally, in every sample of DNA extract from blood, β-actin gene detection was performed in order to check whether rtPCR inhibition takes place; SYBR^®^Green JumpStart Taq ReadyMix (Sigma) was used for that purpose [9].

### DNA Purity and Concentration

The concentration and purity of total DNA extract in the samples were measured spectrophotometrically at wavelengths of *A*_260_ and *A*_280_. Heme concentration in the samples was measured at wavelength of *A*_388_ [4]. The measurement was performed in DNA extracts obtained from whole blood and subjected to preliminary processing. It was performed in a NanoDrop machine (Thermo Scientific).

The contents of microbial DNA in the samples were determined with the help of the rtPCR method using species-specific primers and TaqMan probes (Table 1). Amplification mixture in 10 μL volume: 0.3 U Perpetual *Taq* polymerase (EURx), 200 μM dNTP (EURx), 7.75 mM magnesium chloride (EURx), 200 nM primer (F and R) (Genomed), 300 nM probe (Genomed), and 3 μL DNA. Amplification detection limit was defined as the relation of the *C*_T_ value, i.e., the number of reaction cycle in which the linear increase of the product cuts an established baseline at 30 RFU.

### Evaluation of the rtPCR Method Detection Limit

The evaluation of the PCR method detection limit consisted in simultaneously inoculating the blood samples taken from healthy volunteers with four reference strains simultaneously (*E. coli*, *S. aureus*, *C. albicans*, and *A. fumigatus*), in the same blood sample, so as to obtain a gradient of their number from 10^5^ to 10^0^ CFU/mL—the number of the *A. fumigatus* conidia was determined by counting in a Bürker chamber. Later, DNA was isolated with the use of the prepared methodology and the chosen kit for DNA isolation. The indication of detection limit was performed for three different blood volumes, i.e., 1, 1.5, and 5 mL. DNA was eluted from the columns with the volumes of elution buffer presented in Table 2.

**Table 2:** Volumes of DNA and whole reaction solutions

	Volume of			
	Blood sample volume (mL)	The final DNA eluate (μL)	Amplification reaction (μL)	Template added (μL)
*E. coli*	1	200	10	3
*S. aureus*				
*C. albicans*				
*A. fumigatus*				
*E. coli*	1.5	300	60	30
*S. aureus*				
*C. albicans*				
*A. fumigatus*				
*E. coli*	5	200	10	3
*S. aureus*				
*C. albicans*				
*A. fumigatus*				

The amplification procedure involved 50 cycles

Subsequently, an amplification reaction was conducted with species-specific primers and TaqMan probes (Table 1). This was done individually for each microbe species, in each of their blood dilutions, with the Perpetual *Taq* polymerase (EURx). The procedure of amplification consisted of 50 cycles (Table 1). Amplification mixture: 0.3 U Perpetual *Taq* polymerase (EURx), 200 μM dNTP (EURx), 7.75 mM magnesium chloride (EURx), 200 nM primer (F and R) (Genomed), and 300 nM probe (Genomed). Volumes of DNA solutions (and whole reaction volumes) used are presented in Table 2. DNA isolation and PCR reactions were conducted in five repetitions. Amplification reaction detection limit was determined on the basis of the comparison of parameter *C*_T_ value, i.e., the consecutive reaction cycle number in which the linear increase of the product cut the established baseline at 30 RFU.

### Statistics

In the statistical analysis, Levene’s test was applied—in order to confirm the equality of variances—followed by an ANOVA test. Significant differences were established to be of statistical significance of *P* < 0.05. All calculations were conducted using Gretl software ver. 1.9.4.

## Results

### Preliminary Processing of Blood Samples for Bacterial and Fungal DNA Isolation

As a result of a series of experiments, the following blood-processing procedure was formulated: 6 mL of 0.17 M ammonium chloride (POCh) was added to 1.5 mL of whole blood and incubated at 37 °C for 20 min. Then, the samples were centrifuged at the speed of 10,000 rpm for 10 min and the supernatant was removed. The sediment was suspended in a 100 μL solution of lysozyme (2 mg/mL) (Sigma) and lysostaphin (0.2 mg/mL) (Sigma) in PBS buffer. The whole mixture was transferred to test tubes with glass beads 700–1,100 μm (Sigma) and subjected to mechanical disintegration in FastPrep (MP Biomedicals) machine for 20 s, at speed 4.0 m/s—the mixture was all incubated for 30 min at 37 °C. 200 μL 75 mM NaOH (POCh) was added to the samples and incubated for 10 min at 95 °C. Next, it was centrifuged at speed 12,000 rpm for 10 min and the supernatant was discarded. 500 μL of buffer consisting of 50 mM Tris–HCl pH 7.5 (Sigma), 10 mM EDTA (Sigma), 28 mM β-mercaptoethanol (Sigma), and lyticase 40 U (Sigma) was added to the sediment. The samples were incubated for 30 min at 37 °C and then centrifuged at 12,000 rpm for 10 min. The obtained pellet was submitted to further processing with the use of commercial kits for DNA isolation.

### Evaluation of the Available Commerical Kits for DNA Isolation

DNA was isolated from blood samples subjected to the processing described above with the use of five commercial kits according to the protocols provided by the manufacturers: GeneMATRIX Quick Blood DNA Purification Kit (EURx); GeneJET (Fermentas); QIAamp DNA Blood (QIAGEN); Blood Mini (A&A Biotechnology); and Genomic Mini (A&A Biotechnology).

**Fig. 1:**
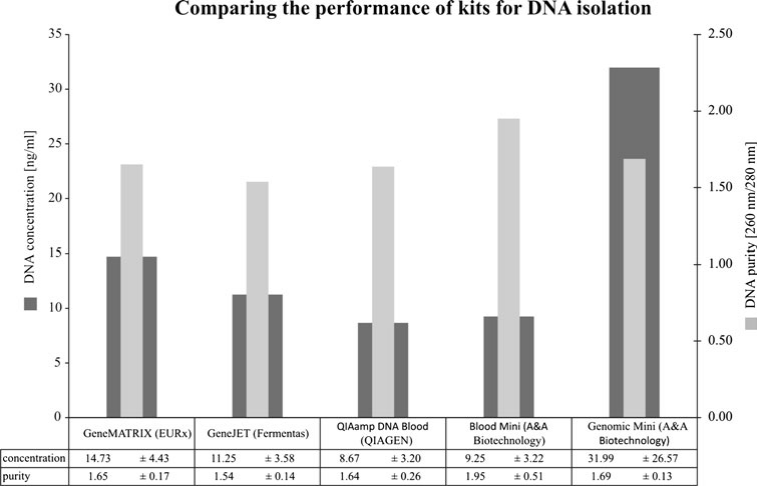
Comparison of kits for isolating DNA from blood as regards the obtained degree of total DNA concentration and its purity, with the application of the developed preliminary blood sample methodology

In the DNA extract obtained, the measurement of the total concentration of DNA and its purity was performed. The highest concentration of deoxyribonucleic acid was found in the isolates obtained with Genomic Mini (A&A Biotechnology) kit—31.99 ng/mL (Fig. 1). The highest purity was noted in DNA extract from the Blood Mini (A&A Biotechnology) set—1.95 ng/mL (Fig. 1). No significant differences were demonstrated between the kits as regards the measured purity and concentration of DNA.

**Fig. 2:**
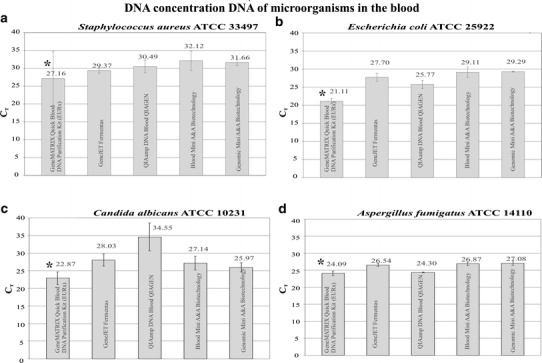
Comparison of DNA content of the microbes: **a** *S. aureus*, **b** *E. coli*, **c** *C. albicans*, and **d** *A. fumigatus* in DNA samples isolated from blood with the application of the elaborated preliminary processing method for blood samples and the studied DNA isolation kits. *Asterisk* values significantly different from the remaining ones for a given microbe (*P* < 0.001)

An evaluation of DNA concentration of the four representatives of the studied microbial species was also performed in the isolated samples. In the case of each set, amplification signal was registered quickly in the DNA samples isolated from blood subjected to preliminary processing. Additionally, GeneMATRIX Quick Blood DNA Purification Kit (EURx) proved to be the best, as the following results were recorded here: *S. aureus* (*C*_T_ = 27.16), *E. coli* (*C*_T_ = 21.11), *C. albicans* (*C*_T_ = 22.87), and *A. fumigatus* (*C*_T_ = 24.09) (Fig. 2a–d). The aforementioned values were significantly different (*P* < 0.001) from the ones obtained with the use of the remaining kits as regards each species. It is an indication that the use of preliminary processing together with the GeneMATRIX Quick Blood DNA Purification Kit (EURx) permits DNA amplification signal to be received faster than from the remaining kits under study.

**Fig. 3:**
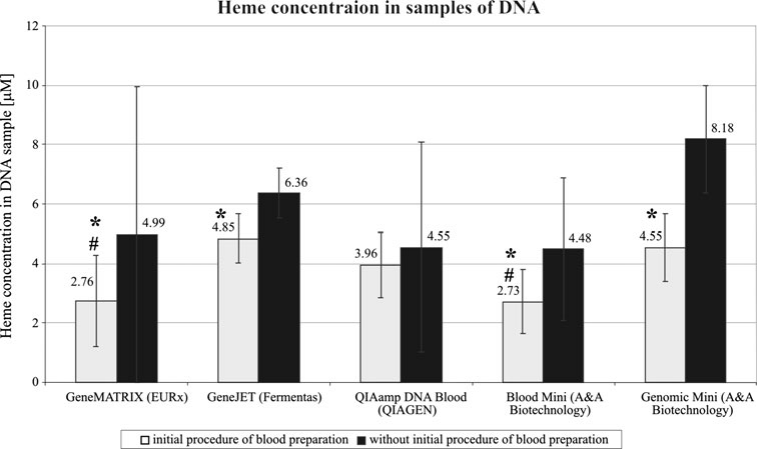
Comparison of the degree of heme concentration in DNA extract obtained with the use of DNA isolation kits both when the developed preliminary blood sample processing was applied and without it. *Asterisk* the significantly different results of heme concentration comparison within one DNA isolation kit when applying preliminary blood samples processing and without it; *Hash* results received by applying preliminary blood samples processing significantly different from the remaining kits under study (*P* < 0.05)

A measurement of heme concentration was also performed in the samples obtained from each of the studied kits for DNA isolation, upon the use of preliminary processing of blood samples or without it. The preliminary procedure allowed the removal of substantially greater (*P* < 0.05) amount of heme than without its application in all of the kits with the exception of QIAamp DNA Blood (QIAGEN), where no significant difference was shown (Fig. 3). The most effective sets as regards removing heme from samples submitted to preliminary processing were: GeneMATRIX Quick Blood DNA Purification Kit (EURx)—2.76 μM and Blood Mini (A&A Biotechnology)—2.73 μM, where the obtained results were significantly (*P* < 0.05) different from the remaining ones, while no significant differences between those kits were shown (Fig. 3). The method omitting preliminary processing did not allow obtaining significant differences between the studied commercial sets. The heme concentration in the blood samples isolated directly with the use of commercial kits yielded from 4.48 to 8.18 μM (Fig. 3).

### Detection Limit of the Studied Microbes with the Use of the rtPCR Method

The minimal limit number of studied microorganisms, the presence of which could be detected in blood during the performed rtPCR reaction, was defined as the method detection limit. A positive result of the amplification reaction indicated that the fluorescence intensity of the PCR product was higher than the one determined for the baseline, at 30 RFU. As regards the DNA samples isolated from 1 to 5 mL of blood, the boundary detection signal for microbial DNA remained at the same level: *E coli*—3.0 × 10^1^ CFU/mL, *S. aureus*—3.0 × 10^3^ CFU/mL, *C. albicans*—3.0 × 10^4^ CFU/mL, and *A. fumigatus* 3.0 × 10^3^ CFU/mL. Isolation from 1.5 mL of blood and the increased volume of elution and DNA content in the reaction mixture allowed raising the determination detection limit per one order of magnitude for *A. fumigatus* (3.0 × 10^2^ CFU/mL) and *E. coli* (3.0 × 10^0^ CFU/mL) and two orders of magnitude for *S. aureus* (3.0 × 10^1^ CFU/mL) and *C. albicans* (3.0 × 10^2^ CFU/mL). The determination was conducted five times and the result was considered significant if the detection signal was received in at least three repetitions.

## Discussion

Detection of microorganisms in blood with the use of the PCR method requires prior isolation of their DNA. Isolation of DNA from leukocytes does not present difficulties as white blood cells are surrounded only by a thin cell membrane. The microbial cell structure brings about the necessity to apply additional processes which could allow digesting their cell walls (e.g., enzymatic lysis) in order to gain access to nucleic acids. The matters get complicated even further because of the fact that blood may contain microorganisms of various cell wall structures, i.e., not only Gram-positive and Gram-negative bacteria, but also yeast or filamentous fungi. Consequently, it is necessary to compile several processes, enabling obtaining high-quality DNA matrix, in order to develop an efficient method for microbial DNA isolation from blood.

In scientific literature, methods for bacterial and fungal DNA isolation from blood are described, but they are enabling one to separately isolate either bacterial or fungal DNA from blood [1, 2, 6, 8, 12].

Also ready-to-use brand name kits for isolating DNA from blood are designed to obtain deoxyribonucleic acid from either only blood or, upon the introduction of an additional stage, from bacteria or from fungi, e.g., QIAamp DNA Blood (QIAGEN) or Blood Mini (A&A Biotechnology). On the market, there are several ready-to-use kits for microbial, both bacterial and fungal, DNA detection from blood, such as Septifast (Roche) or Septitest (Molzym), which enable complex DNA isolation. Yet, there still exists a need for devising new methods of efficient diagnostics of molecular sepsis.

In the present study, a method has been suggested which enables simultaneous DNA isolation of a Gram-negative bacterium *E. coli* ATCC 25922, a Gram-positive one *S. aureus* ATCC 33497, *C. albicans* ATCC 10231 yeast, and a filamentous fungus *A. fumigatus* ATCC 14110 from blood. The four species represent groups of microorganisms which are varied as regards their cell wall structure and can cause sepsis. The developed procedure consisted of three consecutive steps, i.e., mechanical disruption, chemical lysis, and thermal lysis. Afterward, DNA was isolated from the previously prepared samples with the use of several commercial kits for DNA isolation. As was demonstrated in this study, such a procedure allowed obtaining DNA from all four microorganisms under study in the amount sufficient to conduct the rtPCR reaction, which aimed at detecting them in blood. As shown, the lowest sensitivity demonstrated for *A. fumigatus* cells what is associated with composition of its cell wall which is thicker than bacterial cell wall. The GeneMatrix Quick Blood DNA Purification Kit (EURx) proved to be significantly better than the others (Fig. 2a–d). Other authors conducted comparative studies for microbial DNA isolation kits, but they concerned either only bacteria in blood or fungi suspended in an appropriate buffer [6, 7]. The results obtained by them concerned different volumes of blood samples and various isolation kits; moreover, no preliminary processing procedure was applied, so there is no possibility of comparing the results with ours.

The elaborated methodology was also evaluated in terms of the detection limit of *E. coli*, *S. aureus*, *C. albicans*, and *A. fumigatus* depending on the volume of blood samples utilized for DNA isolation. The analysis of the received results permitted to conclude that there is no difference in the detection limit of the method in the case of DNA isolation in 1 and 5 mL volumes; however, the detection limit for *E. coli*, *S. aureus*, *C. albicans*, and *A. fumigates* could be increased by isolating DNA from 1.5 mL of blood and utilizing DNA matrix in the process of amplification in the volume of 30 μL by the mixture volume of 60 μL. This effect is presumably a result of the use of a much larger DNA volume for amplification, which increased the probability of a sequence of microorganism species to appear in a sample in the event of their limit number. The determined method detection limit for detection of the four species allowed to distinguish the number of microbial cells in blood in the quantity of: 3 CFU/mL for *E. coli* and 30 CFU/mL for *S. aureus*, whereas the yeast and the filamentous fungus were characterized by the level of 300 CFU/mL. The obtained results differ from the ones received with the use of SeptiFast (Roche) kit, where they amounted to 3 CFU/mL for *E. coli*, 30 CFU/mL for *S. aureus*, 30 CFU/mL for *C. albicans*, and 3 CFU/mL for *A. fumigatus* [11]. In the case of the fungi, the detection limit determined in this paper is lower, which can be explained by the fact that the methodology presented here assumed the use of 30 μL of DNA as matrix for amplification; while in the case of SeptiFast (Roche) kit, 50 μL was used. The detection limit of detection for both species of bacteria was identical, which probably is connected to the fact that their cell wall gives into lysis much more easily; therefore, it is possible to get more DNA from the same number of cells in comparison to fungi.

Molecular diagnostic of sepsis is a very hard task. It is still the case that blood culture remains the basic diagnostic method, although theoretically there exists technical potential to detect microbes with the use of molecular methods. The difficulties described in this study delimit the direction of essential research, which has to be carried out in order to draw up an efficient and complex method for microbial DNA isolation from the blood.
